# Complete 3-Qubit Grover search on a programmable quantum computer

**DOI:** 10.1038/s41467-017-01904-7

**Published:** 2017-12-04

**Authors:** C. Figgatt, D. Maslov, K. A. Landsman, N. M. Linke, S. Debnath, C. Monroe

**Affiliations:** 1 Joint Quantum Institute, Department of Physics, Joint Center for Quantum Information and Computer Science, University of Maryland, College Park, MD 20742 USA; 2 National Science Foundation, Arlington, VA 22230 USA; 3 IonQ Inc., College Park, MD 20742 USA

## Abstract

The Grover quantum search algorithm is a hallmark application of a quantum computer with a well-known speedup over classical searches of an unsorted database. Here, we report results for a complete three-qubit Grover search algorithm using the scalable quantum computing technology of trapped atomic ions, with better-than-classical performance. Two methods of state marking are used for the oracles: a phase-flip method employed by other experimental demonstrations, and a Boolean method requiring an ancilla qubit that is directly equivalent to the state marking scheme required to perform a classical search. We also report the deterministic implementation of a Toffoli-4 gate, which is used along with Toffoli-3 gates to construct the algorithms; these gates have process fidelities of 70.5% and 89.6%, respectively.

## Abstract (web summary)

Grover’s algorithm provides a quantum speedup when searching through an unsorted database. Here, the authors perform it on 3 qubits using trapped ions, demonstrating two methods for marking the correct result in the algorithm’s oracle and providing data for searches yielding 1 or 2 solutions.

## Introduction

Searching large databases is an important problem with broad applications. The Grover search algorithm^1,2^ provides a powerful method for quantum computers to perform searches with a quadratic speedup in the number of required database queries over classical computers. It is an optimal search algorithm for a quantum computer^3^, and has further applications as a subroutine for other quantum algorithms^4,5^. Searches with two qubits have been demonstrated on a variety of platforms^6–12^ and proposed for others^13^, but larger search spaces have only been demonstrated on a non-scalable NMR system^14^.

**Fig. 1:**
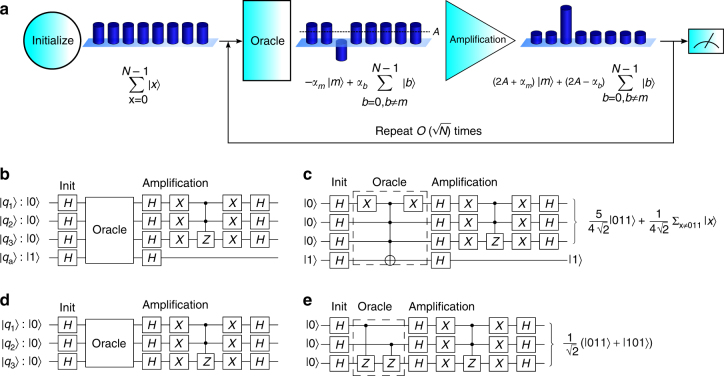
The Grover search algorithm. **a** Evolution of relative amplitudes for each state during a Grover search algorithm. The initialization stage creates an equal superposition of all possible input states, so the amplitude *α*_*x*_ = 1 for all basis states |*x*〉. The oracle stage marks the desired state, so the amplitude *α*_*m*_ of the marked state |*m*〉 becomes negative while the amplitudes *α*_*b*_ of the unmarked states |*b*〉, *b* ≠ *m* remain unchanged. The amplification stage performs a reflection about the mean vector $\mathop {\sum}\nolimits_{x = 0}^{N - 1} {\kern 1pt} \left| x \right\rangle$, which has amplitude $A = {\textstyle{1 \over N}}{\kern 1pt} \mathop {\sum}\nolimits_{x = 0}^{N - 1} {\kern 1pt} \alpha _x = {\textstyle{1 \over N}}\left( { - \alpha _m + \left( {N - 1} \right)\alpha _b} \right)$, to amplify the marked state. An appropriate number of repetitions of the oracle and amplification stages will maximize the amplitude of the correct answer. All qubit states are normalized by the factor ${\textstyle{1 \over {\sqrt N }}}$. The algorithm can also be generalized to mark and amplify the amplitude of *t* desired states. **b** General circuit diagram for a Grover search algorithm using a Boolean oracle, depicted using standard quantum circuit diagram notation^16^. The last qubit *q*_a_ is the ancilla qubit. **c** Example of single-solution Boolean oracle marking the |011〉 state. **d** General circuit diagram for a Grover search algorithm using a phase oracle. **e** Example of two-solution phase oracle marking the |011〉 and |101〉 states

The Grover search algorithm has four stages: initialization, oracle, amplification, and measurement, as shown in Fig. 1a. The initialization stage creates an equal superposition of all states. The oracle stage marks the solution(s) by flipping the sign of that state’s amplitude. The amplification stage performs a reflection about the mean, thus increasing the amplitude of the marked state. Finally, the algorithm output is measured. For a search database of size *N*, the single-shot probability of measuring the correct answer is maximized to near-unity by repeating the oracle and amplification stages *O*((*N*)^1/2^) times^1,2^. By comparison, a classical search algorithm will get the correct answer after an average of *N*/2 queries of the oracle. For large databases, this quadratic speedup represents a significant advantage for quantum computers.

Here, we implement the Grover search algorithm using a scalable trapped atomic ion system^15^ on *n* = 3 qubits, which corresponds to a search database of size *N* = 2^*n*^ = 8. The algorithm is executed for all eight possible single-result oracles and all 28 possible two-result oracles. All searches are performed with a single iteration. For a single-solution algorithm (*t* = 1), the algorithmic probability of measuring the correct state after one iteration is $t \cdot \left( {\left[ {\frac{{N - 2t}}{N} + \frac{{2(N - t)}}{N}} \right]\frac{1}{{\sqrt N }}} \right)^2 = \left( {\frac{5}{{4\sqrt 2 }}} \right)^2 = 78.125\%$ ^2^, compared to $\frac{t}{N} + \frac{{N - t}}{N} \cdot \frac{t}{{N - 1}} = \frac{1}{8} + \frac{7}{8} \cdot \frac{1}{7} = 25\%$ for the optimal classical search strategy, which consists of a single query followed by a random guess in the event the query failed. In the two-solution case (*t* = 2), where two states are marked as correct answers during the oracle stage and both states’ amplitudes are amplified in the algorithm’s amplification stage, the probability of measuring one of the two correct answers is 100% for the quantum case, as compared to $\frac{{13}}{{28}} \approx 46.4\%$ for the classical case. The algorithm is performed with both a phase oracle, which has been previously demonstrated on other experimental systems, and a Boolean oracle, which requires more resources but is directly comparable to a classical search. All quantum solutions are shown to outperform their classical counterparts.

## Results

### Oracles

We examine two alternative methods of encoding the marked state within the oracle. While both methods are mathematically equivalent^16^, only one is directly comparable to a classical search. The Boolean method requires the use of an ancilla qubit initialized to |1〉, as shown in Fig. 1b. The oracle is determined by constructing a circuit out of *NOT* and *C* ^*k*^(*NOT*) (*k* ≤ *n*) gates such that, were the oracle circuit to be implemented classically, the ancilla bit would flip if and only if the input to the circuit is one of the marked states. By using classically available gates, this oracle formulation is directly equivalent to the classical search algorithm, and therefore can most convincingly demonstrate the quantum algorithm’s superiority. On a quantum computer, because the initialization sets up an equal superposition of all possible input states, the *C* ^*n*^(*NOT*) gate targeted on the ancilla provides a phase kickback that flips the phase of the marked state(s) in the data qubits. An example oracle is shown in Fig. 1c to illustrate this. The phase method of oracle implementation does not require the ancilla qubit. Instead, the oracle is implemented with a circuit consisting of *Z* and *C* ^*k*^(*Z*) (*k* ≤ *n* − 1) gates that directly flip the phase(s) of the state(s) to be marked (Fig. 1d, e).

### Experimental setup

The experiments presented here were performed on a programmable quantum computer consisting of a linear chain of five trapped ^171^Yb^+^ ions^17,18^ that are laser cooled near the motional ground state. Qubits are comprised of the first-order magnetic-field-insensitive pair of clock states in the hyperfine-split ^2^ *S*_1/2_ manifold, with |0〉 ≡ |*F* = 0; *m*_*F*_ = 0〉 and |1〉 ≡ |*F* = 1; *m*_*F*_ = 0〉 having a 12.642821 GHz frequency difference. Optical pumping initializes all qubits to the |0〉 state. We execute modular one- and two-qubit gates through Raman transitions driven by a beat note between counter-propagating beams from a pulsed laser^19^, which couples the qubit transition to the collective transverse modes of motion of the ion chain. The qubit–motion interaction provides entangling two-qubit Ising gates^17,20,21^. A pulse segmentation scheme modulates the amplitude and phase of the Raman laser to drive high-fidelity entangling gates using all modes of motion^22,23^. Individual optical addressing of each ion with one Raman beam provides arbitrary single-qubit rotations (*R*(*θ*,*ϕ*)) as well as gates between arbitrary pairs of ions (*XX*(*χ*)) (see Methods for details). State-dependent fluorescence detection with each ion mapped to a separate photomultiplier tube (PMT) channel allows for individual ion readout^15^.

### Toffoli gates

**Fig. 2:**
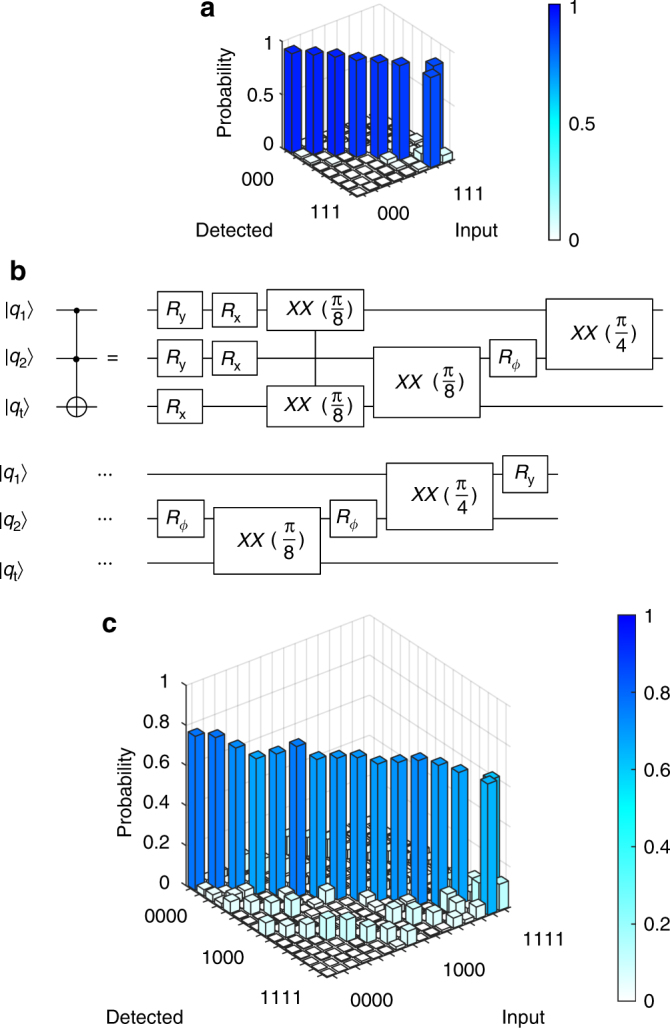
Toffoli implementation. **a** Measured truth table for a Toffoli-3 gate. The average process fidelity is 89.6(2)%, corrected for a 1.5% average state preparation and measurement (SPAM) error. **b** Abbreviated circuit for implementing Toffoli-3 (see Methods for details). **c** Measured truth table for a Toffoli-4 gate performed with three controls, one target, and one ancilla qubit. The average process fidelity is 70.5(3)%, corrected for a 1.9% average SPAM error

Successful demonstration of the Grover search algorithm first requires the implementation of its subroutines. Controlled-NOT (*CNOT*) gates constructed from an $XX\left( {{\textstyle{\pi \over 4}}} \right)$ gate and single-qubit rotations (Methods) have been demonstrated on this system previously^15^. Here, we show results for a controlled-controlled-NOT (*C* ^2^(*NOT*)), or Toffoli-3, gate, with a process fidelity of 89.6(2)% (Fig. 2a). Toffoli-3 gates have been previously performed in NMR systems^24^ and ion traps^25^, including this system^26^. We employed a limited tomography procedure to verify that the Toffoli-3 gate performed had no spurious phases on the outputs (Supplementary Note 1; Supplementary Fig. 1).

Our Toffoli-3 gate is constructed from five two-qubit gates (three $XX\left( {{\textstyle{\pi \over 8}}} \right)$ and two $XX\left( {{\textstyle{\pi \over 4}}} \right)$ gates) in a manner similar to the Toffoli gate demonstrated in ref. ^14^. Any doubly-controlled unitary *C* ^2^(*U*) operation can be performed with five two-qubit interactions (two *CNOT*s, two *C*(*V*)s, and one *C*(*V* ^†^)) if a controlled-*V* operation is available such that *V* ^2^ = *U* ^27^. Since $\left[ {XX\left( {{\textstyle{\pi \over 8}}} \right)} \right]^2 = XX\left( {{\textstyle{\pi \over 4}}} \right)$, we can add single-qubit rotations to construct a Toffoli-3 gate with minimal use of two-qubit gates, as shown in Fig. 2b (see Methods for a detailed circuit diagram). This compares favorably to the six two-qubit gates that would be necessary if only *CNOT* (or equivalently, $XX\left( {{\textstyle{\pi \over 4}}} \right)$) gates were available. These constructions also provide for the implementation of *C*(*Z*) and *C* ^2^(*Z*) gates, which can be constructed by adding a few single-qubit rotations to a *CNOT* or Toffoli-3 gate, respectively (see Methods for circuits). For all circuits, the single-qubit rotations are further optimized to minimize total rotation time^28^.

We use a related strategy to construct a Toffoli-4 gate, and report an average process fidelity of 70.5(3)% (Fig. 2c). Using the methods described in ref. ^29^, we construct a circuit with three control qubits, one target, and one ancilla qubit, requiring 11 two-qubit gates (see Methods for circuit). By again using both $XX\left( {{\textstyle{\pi \over 4}}} \right)$ and $XX\left( {{\textstyle{\pi \over 8}}} \right)$ gates, we are able to save one two-qubit gate relative to a construction limited to *CNOT* gates^29^.

### Data

**Fig. 3:**
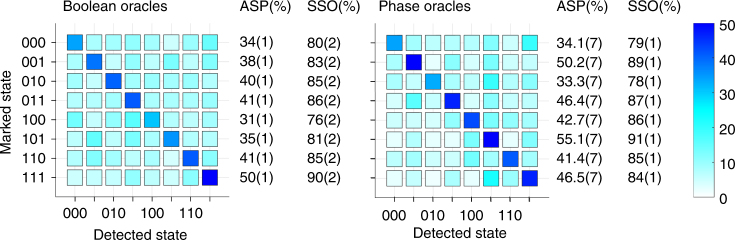
Single-solution algorithm. Results from a single iteration of a single-solution Grover search algorithm performed on a 3-qubit database. Data for the Boolean oracle formulation are shown on the left, and data for the phase oracle formulation are shown on the right. The plots show the probability of detecting each output state. All values shown are percents, with a theoretical ASP of 78.1% and theoretical SSO of 100%. Data are corrected for average SPAM errors of 1%

**Fig. 4:**
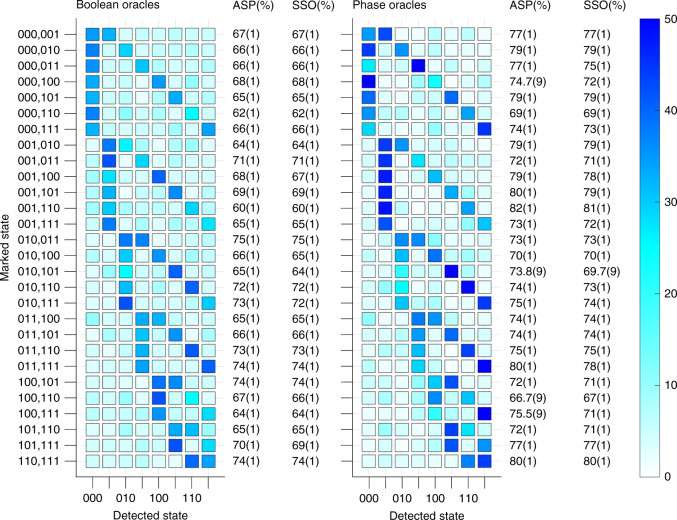
Two-solution algorithm. Results from the execution of a two-solution Grover search algorithm performed on a 3-qubit database. Data for the Boolean oracle formulation are shown on the left, and data for the phase oracle formulation are shown on the right. The plots show the probability of detecting each output state. All values shown are percents. The ASP is the sum of the probabilities of detecting each of the two marked states. Data are corrected for average SPAM errors of 1%

Figures 3 and 4 show the results, respectively, of single- and two-solution Grover search algorithms, each using both the Boolean and phase marking methods (see Methods for optimized circuits performed.). All possible oracles are tested to demonstrate a complete Grover search (Tables 1, 2). Two figures of merit are provided with the data for each oracle. The algorithm success probability (ASP) is the probability of measuring the marked state as the experimental outcome. For the two-solution algorithm, the ASP is calculated by summing the probabilities of measuring each of the two marked states. The squared statistical overlap (SSO) measures the statistical overlap between the measured and expected populations for all states: ${\mathrm{SSO}} = \left( {\mathop {\sum}\nolimits_{j = 0}^N {\kern 1pt} \sqrt {e_jm_j} } \right)^2$, where *e*_*j*_ is the expected population and *m*_*j*_ is the measured population for each state *j* ^30^. Additionally, all of the data shown in this paper is corrected to account for state preparation and measurement (SPAM) errors (see figure captions for values), similar to the method proposed in ref. ^31^ while also accounting for multi-ion crosstalk^15^. All uncertainties given are statistical uncertainties based on the number of experiments performed.

**Table 1:** Single-solution oracles

Table of all oracles used for the single-solution Grover search algorithm

**Table 2:** Two-solution oracles

Table of all oracles used for the two-solution Grover search algorithm

The single iteration, single-solution Grover search algorithm shown in Fig. 3 has a theoretical ASP of 78.1%, as discussed above. The SSO takes into account that the seven unmarked states then have equal expected probabilities totaling 21.9% of being measured. For all Boolean oracles, the average ASP is 38.9(4)% and the average SSO is 83.2(7)%, while phase oracles have an average ASP of 43.7(2)% and an average SSO of 84.9(4)%; the reduced use of resources in the phase oracles (10 *XX*(*χ*) gates and 3 qubits for phase oracles compared to 16 *XX*(*χ*) gates and 5 qubits for Boolean oracles) results in better performance, as expected. These results compare favorably with the classical ASP of 25%.

The two-solution Grover search algorithm shown in Fig. 4 has a theoretical ASP of 100%, as discussed above. For all Boolean oracles, the average ASP is 67.9(2)% and the average SSO is 67.6(2)%, while phase oracles have an average ASP of 75.3(2)% and an average SSO of 74.4(2)%; the reduced use of resources in the phase oracles (6–8 *XX*(*χ*) gates and three qubits for phase oracles compared to 10–14 *XX*(*χ*) gates and four qubits for Boolean oracles) results in better performance, as expected. For all oracles in both cases, the two states with the highest measurement probability are also the two marked states. These results compare favorably with the classical ASP of 46.4%.

### Outlook

We note that this implementation of the Grover search algorithm scales linearly in the two-qubit gate count and ancilla count for increasing search database size as a function of the number of qubits *n*, and for a constant number of solutions *t*. For a database of size *N* = 2^*n*^ stored on *n* qubits, the amplification stage requires one Toffoli-*n* gate, and the *t*-solution oracle stage requires at worst *t* Toffoli-*n* (for a phase oracle) or Toffoli-(*n* + 1) (for a Boolean oracle) gates; optimal oracles for particular sets of marked states may require even fewer two-qubit gates. The method used here to construct the Toffoli-4 circuit scales to Toffoli-*n* gates as 6*n* − 13 in the two-qubit gate count and as $\left\lceil {{\textstyle{{n - 3} \over 2}}} \right\rceil$ in the ancilla count^29^. This paves the way for more extensive use of the Grover search algorithm in solving larger problems on quantum computers, including using the circuit as a subroutine for other quantum algorithms.

## Methods

### Circuit diagrams

Here we present detailed circuit diagrams for all of the operations presented in the paper above, shown in terms of the *R*(*θ*,*ϕ*) and *XX*(*χ*) gates directly implemented by the experiment. The single-qubit rotation is defined as

1 $$R(\theta ,\phi ) = \left( {\begin{array}{*{20}{c}} {{\mathrm{cos}}{\kern 1pt} \frac{\theta }{2}} & { - ie^{ - i\phi }{\kern 1pt} {\mathrm{sin}}{\kern 1pt} \frac{\theta }{2}} \\ { - ie^{i\phi }{\kern 1pt} {\mathrm{sin}}{\kern 1pt} \frac{\theta }{2}} & {{\mathrm{cos}}{\kern 1pt} \frac{\theta }{2}} \end{array}} \right).$$

**Fig. 5:**
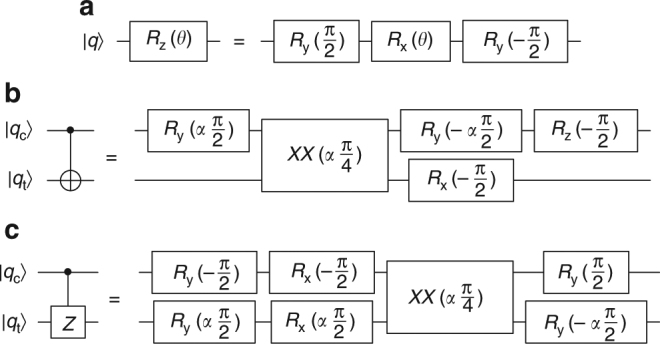
One- and two-qubit composite gates. *χ*_ct_ is the parameter for the *XX* gate between the two qubits. Let *α* = sgn(*χ*_ct_). **a** *R*_*z*_(*θ*) gate implementation using *R*_*x*_(*θ*) and *R*_*y*_(*θ*) gates. **b** *CNOT* gate implementation using *XX*(*χ*), *R*_*x*_(*θ*), *R*_*y*_(*θ*), and *R*_*z*_(*θ*) gates. **c** Controlled-*Z* gate implementation using *XX*(*χ*), *R*_*x*_(*θ*), and *R*_*y*_(*θ*) gates

Rotations about the *X*-axis (*R*_*x*_(*θ*)) are achieved by setting *ϕ* = 0, and rotations about the *Y*-axis (*R*_*y*_(*θ*)) are achieved by setting $\phi = {\textstyle{\pi \over 2}}$. Rotations about the *Z*-axis (*R*_*z*_(*θ*)) are comprised of three rotations about axes in the *XY* plane, as demonstrated in Fig. 5a.

The two-qubit entangling gate is

2 $$XX(\chi ) = \left( {\begin{array}{*{20}{c}} {{\mathrm{cos}}(\chi )} & 0 & 0 & { - i{\kern 1pt} {\mathrm{sin}}(\chi )} \\ 0 & {{\mathrm{cos}}(\chi )} & { - i{\kern 1pt} {\mathrm{sin}}(\chi )} & 0 \\ 0 & { - i{\kern 1pt} {\mathrm{sin}}(\chi )} & {{\mathrm{cos}}(\chi )} & 0 \\ { - i{\kern 1pt} {\mathrm{sin}}(\chi )} & 0 & 0 & {{\mathrm{cos}}(\chi )} \end{array}} \right).$$

The parameter *χ* can be varied continuously by adjusting the overall power applied to the gate, but the gates used here require only $\chi = \pm {\textstyle{\pi \over 4}}$ or $\chi = \pm {\textstyle{\pi \over 8}}$. The gate is maximally entangling for $\chi = \pm {\textstyle{\pi \over 4}}$, so $XX\left( {{\textstyle{\pi \over 4}}} \right)\left| {00} \right\rangle = {\textstyle{1 \over {\sqrt 2 }}}\left( {\left| {00} \right\rangle - i\left| {11} \right\rangle } \right)$.

Two-qubit *XX* gates are combined with rotation *R* gates to construct the composite gates needed for the Grover search algorithm implementation. The parameter *χ* can be positive or negative, depending on what ion pair is chosen and the particulars of the pulse segmentation solution chosen for the ion pair in question; the sign of *χ* (sgn(*χ*)) is determined experimentally for each ion pair. Consequently, some composite gate circuits include rotations with parameters that depend on sgn(*χ*). Composite gates were constructed by starting with known circuits, converting constituent parts into *R* and *XX* gates using lower-level constructions, and then optimizing the circuit. First, the number of *XX* gates was minimized (as in the Toffoli-3 gate, described in the main text). Second, the single-qubit gates were optimized by minimizing the sum of all rotation angles *θ*, as this minimizes the total time for the experiment. Additional details can be found in refs. ^28,29^.

**Fig. 6:**
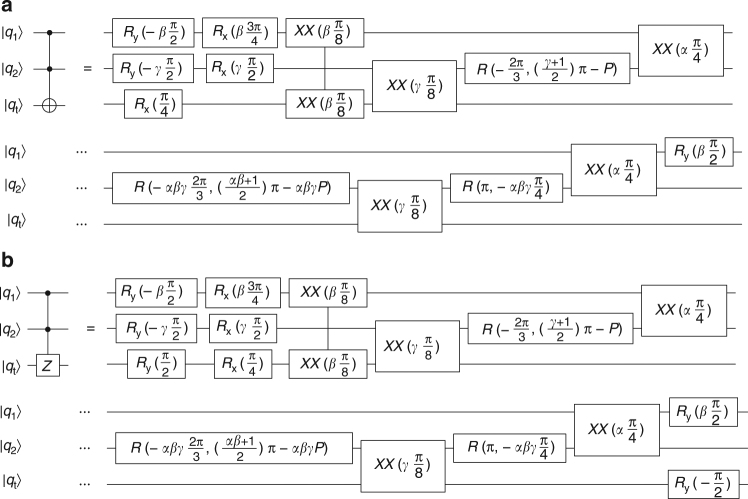
Three-qubit composite gates. Three-qubit composite gates using *XX*(*χ*), *R*_*x*_(*θ*), *R*_*y*_(*θ*), and *R*(*θ*,*ϕ*) gates. Let *α* = sgn(*χ*_12_), *β* = sgn(*χ*_1t_), *γ* = sgn(*χ*_2t_), and $P = {\mathrm{arcsin}}{\kern 1pt} \left( {\sqrt {{\textstyle{2 \over 3}}} } \right)$. **a** Toffoli-3 gate implementation. **b** Controlled-controlled-*Z* (*CCZ*) gate implementation

**Fig. 7:**
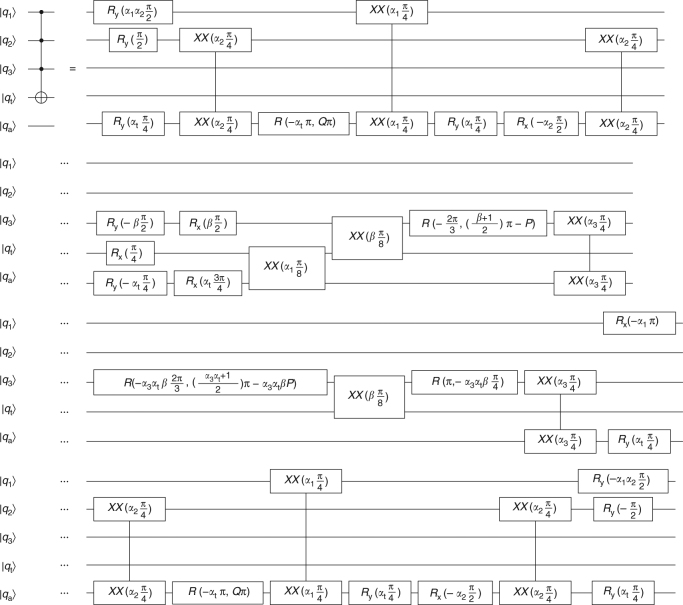
Toffoli-4 gate. Toffoli-4 gate implementation using *XX*(*χ*), *R*_*x*_(*θ*), *R*_*y*_(*θ*), and *R*(*θ*, *ϕ*) gates. Let *α*_1_ = sgn(*χ*_1a_), *α*_2_ = sgn(*χ*_2a_), *α*_3_ = sgn(*χ*_3a_), *α*_t_ = sgn(*χ*_ta_), *β* = sgn(*χ*_3t_), $P = {\mathrm{arcsin}}{\kern 1pt} \left( {\sqrt {{\textstyle{2 \over 3}}} } \right)$, and $Q = {\textstyle{1 \over 8}}\left( {4 - 3\alpha _2\alpha _{\mathrm{t}}} \right)$

The two-qubit *CNOT* and controlled-*Z* gates are shown in Fig. 5b, c. They each require one *XX* gate and several rotations. The three-qubit gates used here are the Toffoli-3 and controlled-controlled-*Z* (*CCZ*) gates, shown in Fig. 6a, b. The Toffoli-3 gate requires two control qubits (*q*_1_ and *q*_2_) and one target qubit (*q*_t_). Finally, the four-qubit Toffoli-4 gate is shown in Fig. 7. It governs a four-qubit interaction between three control qubits (*q*_1_, *q*_2_, and *q*_3_) and one target qubit (*q*_t_), and it additionally requires an ancilla qubit (*q*_a_).

**Fig. 8:**
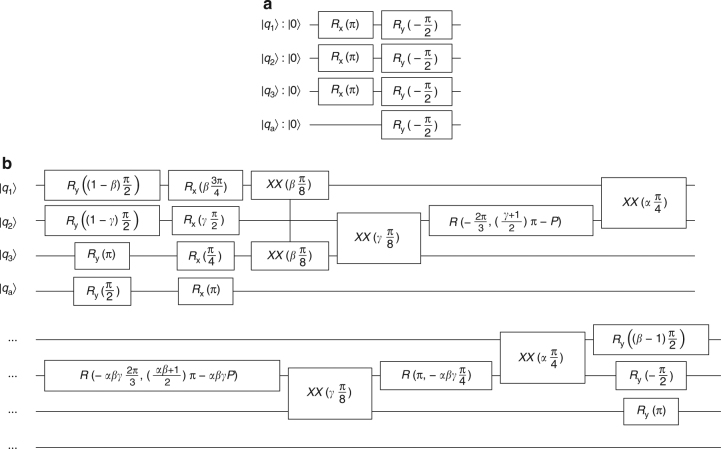
Grover search algorithm circuits. Grover search algorithm implementation by substage using *XX*(*χ*), *R*_*x*_(*θ*), *R*_*y*_(*θ*), and *R*(*θ*, *ϕ*) gates. The circuits shown are for use with Boolean oracles; removing the ancilla qubit |*q*_a_〉 produces the necessary circuits for use with a phase oracle. Let *α* = sgn(*χ*_12_), *β* = sgn(*χ*_1t_), *γ* = sgn(*χ*_2t_), and $P = {\mathrm{arcsin}}{\kern 1pt} \left( {\sqrt {{\textstyle{2 \over 3}}} } \right)$. **a** Grover initialization stage implementation. **b** Grover amplification stage implementation

The Grover search algorithm is implemented using circuits that are equivalent to those shown in Fig. 1b, d, but with the initialization and amplification stages optimized to minimize gate times, as shown in Fig. 8a, b. The circuits shown are for use with Boolean oracles; in the phase oracle case, the ancilla qubit *q*_a_ is simply omitted. To preserve the modularity of the algorithm, the initialization stage and amplification stage were each optimized without regard to the contents of the oracle, so each possible oracle can simply be inserted into the algorithm without making any changes to the other stages.

Oracles for the Grover search algorithm were constructed using a combination of reversible and classical logic synthesis techniques. For Boolean oracles, reversible logic synthesis was employed to find a set of *X*, *C* ^*N*^(*NOT*) gates that marked the desired state(s) for each oracle. For phase oracles, EXOR polynomial synthesis was used to find a set of *Z*, *C* ^*N*^(*Z*) gates that marked the desired state(s) for each oracle. For example, for Boolean oracles, the selection was limited to the classically available *X* (or *NOT*) and *C* ^*N*^(*NOT*) gates, and a reversible circuit was constructed such that the output bit (corresponding to the ancilla qubit in the quantum oracle) would be flipped if and only if a marked state was used as the input to the circuit. While there are many possible circuit constructions for each oracle, the oracle chosen for implementation was one that first minimized the number of two-qubit interactions required, and then minimized the number of single-qubit interactions needed. The synthesis techniques used are scalable and can be applied to oracles of any size. The oracles used here were implemented as per the circuit diagrams shown in Table 1 for single-solution oracles and Table 2 for two-solution oracles.

Other quantum algorithms may be implemented on this system in a similar fashion. First, decompose the algorithm’s subroutines into high-level circuits. Second, optimize those circuits to minimize the number of two-qubit interactions required. Third, decompose the high-level circuits into physical-level *R* and *XX* gates. Finally, perform further optimizations to first minimize the number of two-qubit *XX* gates required, and then to minimize the total rotation time (the sum of all rotation angles *θ*) across all *R* gates. However, since the optimization of quantum circuits is QMA-Hard, we anticipate that future improvements in algorithm design, circuit synthesis, and circuit optimization techniques may result in more efficient circuit implementations, facilitating increased experimental performance.

### Data availability

All relevant data are available from the corresponding author upon request.

## Electronic supplementary material

Supplementary Information
