# Supplementary material for: Complete 3-Qubit Grover search on a programmable quantum computer
Source: Nat Commun. 2017 Dec 4;8:1918. doi: 10.1038/s41467-017-01904-7 (PMC5715115; doi:10.1038/s41467-017-01904-7)
Supplement: Supplementary file 1 — Supplementary Information [file 41467_2017_1904_MOESM1_ESM.pdf]

SUPPLEMENTARY NOTE 1

Experimental Error Sources The measured process fidelities are reduced from the theoretical ideal fidelities primarily through technical imperfections in the experimental system. The predominant source of error is beam pointing instability on the individual Raman beams, causing laser intensity fluctuations at the ions. This results in small random coherent errors. Other error sources include inhomogeneous Stark shifts across the ion chain that could not be perfectly compensated, and crosstalk between individual ion controls. These control problems can largely be solved through improved engineering of key components. Future work on the experiment will include individual phase and frequency control over each individual Raman beam as well as active beam steering and power stabilization. Improvements in multizone, microfabricated ion trap technology provide control over tens of ions in a single trap, and further scalability may be achieved by connecting multiple ion trap modules through ion shuttling [1] or photonic interconnects [2].

Additional Iterations Performing an additional iteration on the single-solution Grover search algorithms was inhibited by circuit-depth limitations in the experimental control program, which will be fixed for future work. Here, we estimate the impact of an additional iteration on algorithm performance. While a single iteration of the single-solution Grover search algorithm has a maximum ASP of 78.125%, performing two iterations raises the maximum ASP to 94.5312%. Applying the error estimation models used in [3], the likely performances of two Grover search algorithm cases were examined: the Boolean 000 oracle and the phase 111 oracle, which correspond to the worst- and best-case oracles by gate count. The random error estimation model assumes random error propagation for each operation of the form $(1-\epsilon_{\mathrm{g}})^{\sqrt{N}}$, and the systematic error estimation model assumes coherent over- or under-rotations for each operation and has the form $(1-\epsilon_{\mathrm{g}})^{N}$, where N is the number of operations and ϵ_{g} is the error per operation. Based on the analysis in [3] on this same system, we expect the actual results to fall somewhere between these two models. For a single iteration of the phase 111 oracle, we estimate an SSO of 86% and an ASP of 41% using the random error model, or an SSO of 61% and an ASP of 16% using the systematic error model; as in the analysis in [3], we compare this to the measured SSO of 84(1)% and ASP of 46.5(7)% and see that the experiment performs slightly worse than the random error model, and better than the systematic error model. Extending the analysis to two iterations of the phase 111 oracle, we estimate an SSO of 81% and ASP of 60% using the random error model, or an SSO of 40% and an ASP of 19% using the systematic error model.

Similarly, for a single iteration of the Boolean 000 oracle, we estimate an SSO of 83% and an ASP of 37% using the random error model, or an SSO of 45% and an ASP of 6% using the systematic error model; as before, we compare this to the measured SSO of 80(2)% and ASP of 34(1)% and see that the experiment performs slightly worse than the random error model. Extending the analysis to two iterations of the phase 111 oracle, we estimate an SSO of 77% and ASP of 55% using the random error model, or an SSO of 22% and an ASP of 6% using the systematic error model. We expect the experiment would perform somewhere between these two models, although we do not know how well these error models hold for very deep circuits; it is not clear whether the experiment would have outperformed the best classical strategy with two iterations, which has a success probability of 37.5%.

Toffoli-3 Characterization We employed a limited tomography procedure to characterize the outputs of the Toffoli-3 gate performed. A global rotation into the X basis was applied to all 3 ions before and after the Toffoli-3 gate for each input (see Figure 1(a)): $R_{\mathrm{y}}(\frac{\pi}{2})$ for the even inputs (000, 010, 100, 110) and $R_{\mathrm{y}}(-\frac{\pi}{2})$ for the odd inputs (001, 011, 101, 111). An ideal Toffoli-3 gate will result in an anti-diagonal input-output matrix in the Z basis when this procedure is applied. The experimental results of this verification procedure are shown in Figure 1(b) with an average success probability of 82.1(2)%, indicating the Toffoli-3 is faithful for arbitrary input states.

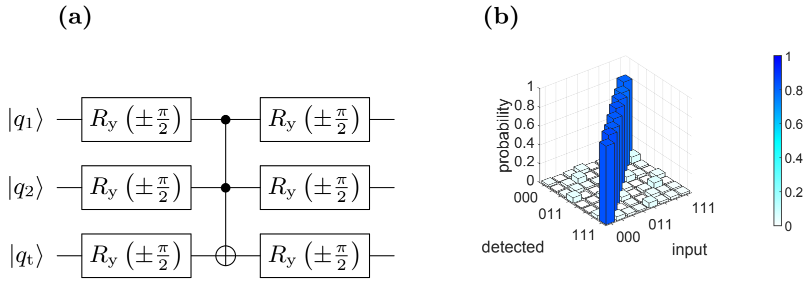

Supplementary Figure 1. **Toffoli-3 characterization.** (a) Circuit for implementing the Toffoli-3 limited tomography procedure. The global rotations are positive for even input states and negative for odd input states. (b) Limited tomography check performed on the Toffoli-3 gate to verify phases. The average success probability is 82.1(2)%, corrected for a 2.4% average SPAM error.

[1] Kielpinski, D., Monroe, C. & Wineland, D. J. Architecture for a large-scale ion-trap quantum computer. *Nature* **417**, 709–711 (2002).

[2] Monroe, C. *et al.* Large-scale modular quantum-computer architecture with atomic memory and photonic interconnects. *Phys. Rev. A* **89**, 022317 (2014).

[3] Linke, N. M. *et al.* Experimental comparison of two quantum computing architectures. *PNAS* **114**, 3305–3310 (2017).
